# An AI-Powered Precision Patient Tracking System for Interdepartmental Patient Identification and Recall: A Real-World Longitudinal Study

**DOI:** 10.3390/healthcare14213586

**Published:** 2026-10-23

**Authors:** Jiaman Liao, Changhai Wu, Yusong Liu, Yiqiang Zhan

**Affiliations:** 1 Operations Management Office, Longgang Central Hospital of Shenzhen, Shenzhen 518116, China; 2 Department of Epidemiology, School of Public Health (Shenzhen), Sun Yat-Sen University, Shenzhen 518107, China

**Keywords:** artificial intelligence, patient tracking, patient recall, cross-department follow-up, care coordination, digital health

## Abstract

**Background**: During specialty care, clinical assessments may identify abnormalities requiring evaluation by another department. However, gaps in cross-department follow-up may delay subsequent specialty evaluation. This study evaluated the application of an AI-Powered Precision Patient Tracking System (APTS) for cross-department patient identification, recall, and return-to-care tracking and examined its association with changes in departmental outpatient volume. **Methods**: We conducted a single-center longitudinal study using monthly data from nine clinical departments at Shenzhen Longgang Central Hospital between January 2025 and September 2026, comprising 189 department-month observations. APTS integrates multisource clinical information to support patient identification, task assignment, recall management, and return-to-care tracking. The Callaway–Sant’Anna staggered difference-in-differences approach was used to estimate changes in monthly unique outpatients and outpatient visits following APTS implementation. APTS operational measures and the departmental origins of patients returning to outpatient care following recall were also analyzed. **Results**: Following APTS implementation, 109,620 patients were assigned for subsequent management, of whom 50,429 (46.0%) entered recall management and 9525 returned to outpatient care following APTS recall. Of these 9525 patients, 8593 (90.2%) originated from departments other than the target department, with such patients constituting the majority in all nine departments. APTS implementation was not significantly associated with changes in monthly unique outpatients (−2.5%; 95% CI, −11.2% to 7.0%) or outpatient visits (−1.9%; 95% CI, −12.1% to 9.5%). **Conclusions**: The potential value of APTS may lie in supporting the identification and management of patients across departmental boundaries. No statistically significant change in departmental outpatient volume was detected within the available identification window, although potentially meaningful changes could not be excluded.

## 1. Introduction

With the advancement of tiered healthcare systems and strategies for high-quality hospital development [1,2,3], timely identification and follow-up of patients with clinically significant abnormal test results have become important components of patient safety and continuity of care [4,5]. Meanwhile, the digital transformation of healthcare provides opportunities to improve the organization and coordination of clinical services [6,7], particularly the efficiency, timeliness, and continuity of patient follow-up [8].

During specialty care, laboratory tests, imaging studies, or other clinical assessments may reveal abnormalities that require further evaluation and management by another specialty. However, patient identification, referral, and follow-up are often fragmented across departments and information systems [9,10]. Such gaps may be particularly relevant when the department identifying an abnormality differs from the specialty responsible for subsequent management. Therefore, timely identification of these patients and their continued tracking to the appropriate specialty represent important challenges in hospital-based patient management.

Traditional patient follow-up relies largely on manual screening, referral, and follow-up procedures, resulting in substantial workloads and making it difficult to establish continuous and traceable management pathways across departments. In recent years, telemedicine [11,12,13], patient tracking, and digital follow-up technologies [14,15,16] have increasingly been incorporated into patient management. However, many existing approaches focus on individual stages of the care pathway rather than integrating patient identification, task assignment, active recall, and return-to-care tracking into a structured management pathway. This limitation is particularly relevant when abnormalities are identified outside the specialty ultimately responsible for subsequent care. Effective management in such situations requires not only identifying patients who need further specialty evaluation but also directing them to the appropriate department and tracking their subsequent care.

Recent studies have highlighted the expanding role of artificial intelligence (AI) in healthcare innovation, including advances in rehabilitation technology transfer and collaborative innovation in AI medical devices [17,18]. These developments underscore the growing integration of AI technologies into healthcare systems and the increasing involvement of medical institutions in technological innovation. In clinical practice, AI also offers the capacity to extract and integrate information from heterogeneous clinical data and support patient screening and task assignment, providing a potential approach to establishing structured management pathways. However, previous applications of AI in healthcare have predominantly focused on algorithm development, diagnostic performance, and risk prediction, whereas real-world evidence regarding AI-supported continuous patient management within routine clinical workflows remains limited. In particular, when patients requiring further specialty management are distributed across multiple clinical departments, evidence remains insufficient regarding how AI-powered tracking systems can support structured cross-department pathways for patient identification, recall, and return-to-care tracking. Moreover, whether the implementation of such systems is associated with changes in departmental outpatient volume remains unclear.

Accordingly, this study used a single-center longitudinal design with staggered implementation across multiple clinical departments to evaluate the real-world application of an AI-Powered Precision Patient Tracking System (APTS). By integrating multisource clinical information, APTS supports a structured management pathway encompassing patient identification, task assignment, recall management, and return-to-care tracking. We characterized patient assignment, recall management, and return to outpatient care following APTS implementation and examined the departmental origins of recalled patients to assess the system’s role in cross-department patient follow-up. In parallel, we applied the Callaway–Sant’Anna staggered difference-in-differences approach to evaluate the association between APTS implementation and changes in departmental outpatient volume, thereby further characterizing the role of APTS in routine clinical patient management.

## 2. Materials and Methods

### 2.1. Study Setting, APTS, and Data Sources

#### 2.1.1. APTS Overview

APTS integrates the Qizhen medical large language model (QizhenGPT-32B; Institute of Computer Innovation and Technology, Zhejiang University, Hangzhou, China), used through an institutional collaboration between Shenzhen Longgang Central Hospital and the Institute of Computer Innovation and Technology of Zhejiang University, with the hospital’s clinical information systems to support the identification and management of patients who may require further diagnostic evaluation, treatment, or follow-up. The system integrates multisource clinical information, including laboratory test results, imaging findings, electronic health records, and other available clinical data. AI-assisted screening is performed according to configurable, department-specific management rules, including screening criteria, alert levels, target departments, and designated case managers. The large language model supports the semantic interpretation of clinical information, including unstructured clinical text, to identify patients meeting the configured screening criteria. These criteria may incorporate numerical thresholds, supplementary clinical knowledge, and comparisons with historical records.

Once a patient is identified, the system assigns the corresponding management task to the appropriate target department and case manager. The designated case manager then manually reviews the assigned patient information to determine whether active recall is required. Only patients confirmed as requiring active recall enter recall management. Patients entering recall management may subsequently be contacted by telephone, text message, the hospital’s official WeChat account, or other available communication channels and guided toward appropriate follow-up care. APTS records recall management and return-to-care activities, thereby integrating patient identification, task assignment, manual review, recall management, and return-to-care tracking into a structured management pathway. The overall APTS workflow is illustrated in Figure 1.

APTS is designed to support, rather than replace, clinical decision-making. Clinical interpretation and decisions regarding subsequent patient management remain the responsibility of the relevant clinical teams.

#### 2.1.2. Data Sources and Study Design

This was a single-center, real-world longitudinal study evaluating the implementation of APTS across multiple clinical departments. The study period extended from January 2025 to September 2026, comprising 21 consecutive calendar months. Nine clinical departments with complete monthly observations throughout the study period were included, yielding a total of 189 department-month observations.

APTS was implemented sequentially across departments at different calendar times, resulting in a staggered implementation design. Implementation began in March 2025, and all included departments had implemented APTS by October 2025. For each department, the month of formal APTS implementation was defined as the first post-implementation month.

Department-level monthly data included outpatient visits, unique outpatients, patients assigned by APTS, patients entering recall management, patients returning to outpatient care following APTS recall (return-to-care patients), and the departmental origins of recalled outpatients. APTS-specific operational measures were derived from structured system management records.

#### 2.1.3. Study Departments and Unit of Analysis

The primary unit of analysis was the department-month. Each of the nine included departments contributed 21 consecutive monthly observations, yielding a total of 189 department-month observations. These longitudinal department-month data were used to evaluate changes in departmental outpatient volume.

In addition, APTS operational measures were summarized over the post-implementation period to characterize system activity, including patient assignment, recall management, return to outpatient care following APTS recall, and the departmental origins of recalled outpatients. These operational measures were summarized descriptively and were not used to estimate patient-level treatment effects.

### 2.2. Outcome Variables

#### 2.2.1. APTS Implementation

For each department, the month of formal APTS implementation was defined as the first treated month. Department-month observations before implementation were considered untreated, whereas the implementation month and all subsequent months were considered treated. Because implementation timing differed across departments, treatment cohorts were defined according to each department’s first implementation month.

#### 2.2.2. Department-Level Outcome Measures

The primary department-level outcome was the number of monthly unique outpatients, defined as the number of distinct patients receiving outpatient care in each department during each calendar month. This outcome was used to assess whether APTS implementation was associated with changes in the number of patients receiving outpatient care in the target departments. The secondary outcome was the number of monthly outpatient visits, reflecting the overall volume of outpatient services provided by each department.

Given substantial differences in department size and baseline outpatient volume across the nine departments, both outcomes were log-transformed for the primary difference-in-differences analysis. Estimates on the logarithmic scale were subsequently converted to relative percentage changes using the formula 100 × [exp(ATT) − 1].

#### 2.2.3. APTS Operational Measures

To characterize APTS activity following implementation, three APTS-specific operational measures were summarized: (1) patients assigned by APTS, defined as patients assigned through APTS for subsequent management by the target department; (2) patients entering recall management, defined as patients assigned by APTS who were confirmed through manual review by the designated case managers as requiring active recall and subsequently entered the recall management process; and (3) patients returning to outpatient care following APTS recall, defined as patients who returned to the target department after recall management through telephone calls, text messages, or the hospital’s official WeChat account. Return visits were identified by linking APTS recall management records with outpatient records using a unique patient identifier, which remained consistent across outpatient encounters. Patients were counted if they returned after recall management and before the data extraction cutoff, without a fixed follow-up window.

The proportion entering recall management was calculated as the number of patients entering recall management divided by the number of patients assigned by APTS. The recall–return rate was calculated as the number of return-to-care patients divided by the number of patients entering recall management. Both proportions were calculated from aggregated patient counts over the post-implementation period rather than as averages of monthly or department-specific proportions.

#### 2.2.4. Departmental Origins of Recalled Outpatients

The source department was defined as the department where the patient was initially identified by APTS, whereas the target department was the department assigned for subsequent management. Return-to-care patients were classified according to whether their source department was the same as the target department responsible for subsequent management. Patients whose source department was the same as the target department were classified as originating from the target department, whereas those whose source department differed from the target department were classified as originating from other departments. The latter group represented patients identified from outside the target department and subsequently incorporated into its recall and follow-up management pathway.

### 2.3. Statistical Analysis

#### 2.3.1. Descriptive Statistics

Descriptive analyses were conducted for all department-month observations. Because APTS-related operational measures, including patient assignment, recall management, and return to outpatient care following APTS recall, exhibited right-skewed distributions, continuous variables were summarized using medians and interquartile ranges (IQRs), whereas categorical variables were summarized using frequencies and percentages.

#### 2.3.2. Staggered Difference-in-Differences Analysis

The Callaway–Sant’Anna staggered difference-in-differences approach was used to evaluate changes in departmental outpatient volume following APTS implementation while accounting for variation in implementation timing across departments. At each calendar time point, departments that had not yet implemented APTS served as the comparison group for departments in which APTS had already been implemented. Because all included departments eventually implemented APTS, no never-treated comparison group was available. Because all nine departments had implemented APTS by October 2025, not-yet-treated comparison departments were available only through September 2025. Accordingly, the analysis yielded 19 identifiable post-implementation group-time treatment effect estimates across calendar months from March to September 2025, corresponding to event times 0 to +6. No post-implementation treatment effects could be identified from October 2025 onward using not-yet-treated comparisons. The remaining longitudinal observations were retained for descriptive analyses.

A doubly robust estimator was used to estimate group-time average treatment effects, which were subsequently aggregated to obtain the overall average treatment effect on the treated (ATT). In the primary analysis, monthly unique outpatients and monthly outpatient visits were log-transformed to improve comparability across departments with substantial differences in baseline outpatient volume. ATT estimates on the logarithmic scale were converted to relative percentage changes using the formula 100 × [exp(ATT) − 1].

Given the inclusion of only nine departments and the decreasing number of not-yet-treated departments available as comparisons as implementation progressed, interpretation emphasized effect estimates and confidence intervals rather than statistical significance alone.

#### 2.3.3. Event-Time Analysis

Dynamic effects associated with APTS implementation were assessed using event-time aggregation of the Callaway–Sant’Anna group-time ATT estimates. Event time was defined relative to the month of APTS implementation, with event time 0 representing the month of implementation. In the main figures, estimates from 4 months before implementation to 3 months after implementation were presented to focus on event times with relatively greater support across treatment cohorts. The full event-time analysis produced pre-implementation estimates for event times −7 to −1 and post-implementation estimates for event times 0 to +6. However, the main figure was restricted to event times −4 to +3, with later post-implementation event times supported by fewer implementation cohorts.

Event-time effects were estimated on the logarithmic scale and subsequently converted to percentage changes. To account for multiple comparisons across event times, 95% simultaneous confidence bands were constructed using the multiplier bootstrap. Pre-implementation event-time estimates were also examined to assess evidence of differential pre-implementation trends. Given the limited number of departments and the relatively short pre-implementation periods available for some early-implementing departments, assessment of the parallel trends assumption should be interpreted with caution.

#### 2.3.4. Sensitivity Analysis

As a sensitivity analysis, the staggered difference-in-differences model was re-estimated using monthly unique outpatients and monthly outpatient visits on the original count scale rather than the log-transformed scale. This analysis was conducted to assess whether the findings were sensitive to the scale used for the outcome measures.

#### 2.3.5. Statistical Software

All statistical analyses were performed using R software (version 4.5.1). The Callaway–Sant’Anna staggered difference-in-differences analysis was implemented using the R package did (version 2.3.0) [19,20]. All statistical tests were two-sided, and a two-sided *p*-value < 0.05 was considered statistically significant.

## 3. Results

### 3.1. Study Sample and Department Characteristics

Nine clinical departments were included and observed monthly from January 2025 to September 2026, yielding a total of 189 department-month observations. APTS was implemented sequentially across departments between March and October 2025. The number of pre-implementation observation months ranged from 2 to 9 across departments, whereas the number of post-implementation observation months ranged from 12 to 19.

Substantial differences in outpatient volume were observed across departments. For example, the median monthly number of unique outpatients was 156 in Cardiothoracic Surgery and 4226 in General Surgery, while the corresponding median numbers of monthly outpatient visits were 161 and 4532, respectively. Department characteristics and APTS implementation timing are summarized in Table 1.

### 3.2. Changes in Department-Level Outpatient Volume Following APTS Implementation

In the staggered difference-in-differences analysis, APTS implementation was not significantly associated with changes in departmental outpatient volume. For the primary outcome, the estimated change in monthly unique outpatients was −2.5% (95% CI, −11.2% to 7.0%). For the secondary outcome, the estimated change in monthly outpatient visits was −1.9% (95% CI, −12.1% to 9.5%) (Table 2). These estimates were based on identifiable group-time comparisons from March to September 2025, rather than the entire longitudinal observation period through September 2026.

### 3.3. Event-Time Analysis of Department-Level Outpatient Volume

Event-time estimates for monthly unique outpatients showed no statistically significant pre-implementation deviations from zero. However, given the limited number of departments and the relatively short pre-implementation periods available for some early-implementing departments, these findings were insufficient to establish the parallel trends assumption (Figure 2). Following APTS implementation, point estimates suggested small increases in monthly unique outpatients, with estimated changes of approximately 3.1% in the implementation month and 3.7% one month after implementation. However, the 95% simultaneous confidence bands included zero at all event times.

Point estimates subsequently moved toward zero and became negative at later event times; however, none of the post-implementation event-time estimates differed significantly from zero based on the simultaneous confidence bands. Overall, the event-time analysis provided no evidence of a sustained change in monthly unique outpatients following APTS implementation.

### 3.4. APTS Operational Measures and Return-to-Care

Following APTS implementation, 109,620 patients were assigned by APTS for subsequent management. Following manual review by designated case managers, 50,429 (46.0%) were confirmed as requiring active recall and entered recall management, of whom 9525 subsequently returned to outpatient care following APTS recall. The overall recall–return rate was 18.9% (Table 3). The proportion of assigned patients entering recall management varied substantially across departments, ranging from 4.3% to 81.8%.

### 3.5. Departmental Origins of Patients Returning to Outpatient Care Following APTS Recall

Among the 9525 patients who returned to outpatient care following APTS recall, 8593 (90.2%) originated from departments other than the target department, whereas 932 (9.8%) originated from the target department. Patients originating from other departments constituted the majority of recalled outpatients in all nine departments, with proportions ranging from 68.4% to 100.0% (Figure 3 and Supplementary Table S1).

The proportion originating from other departments was highest in Endocrinology (100.0%), Nephrology (96.9%), Urology (96.5%), and Infectious Diseases (96.1%). Overall, the predominance of patients originating from other departments reflects the predefined patient identification and assignment rules of APTS.

### 3.6. Sensitivity Analysis Using the Original Count Scale

When the staggered difference-in-differences model was re-estimated using the original count scale, the findings were consistent with those of the primary analysis, with all 95% CIs including zero. APTS implementation was associated with an estimated increase of 56.6 monthly unique outpatients (95% CI, −403.7 to 517.0) and 73.1 monthly outpatient visits (95% CI, −450.7 to 596.9); neither estimate was statistically significant (Supplementary Table S2). Given the substantial differences in baseline outpatient volume across departments, the count-scale estimates had wide confidence intervals. Overall, the sensitivity analysis provided no evidence that the primary findings were materially dependent on the scale used for the outcome measures.

## 4. Discussion

In this real-world longitudinal study, we evaluated the phased implementation of APTS across nine clinical departments. The most notable finding was that, among the 9525 patients who returned to outpatient care following APTS recall, 90.2% originated from departments other than the target department, and patients from other departments constituted the majority in all nine departments. APTS also supported a structured management pathway encompassing patient identification, task assignment, recall management, and return-to-care tracking. Overall, 109,620 patients were assigned by APTS for subsequent management, of whom 50,429 entered recall management. Despite this substantial volume of patient management activity, APTS implementation was not associated with significant changes in departmental outpatient volume. Taken together, these findings suggest that the potential value of APTS may lie less in expanding outpatient service volume than in identifying patients outside the target department who require further specialty management and incorporating them into a structured, cross-departmental management pathway.

The predominance of patients originating from departments other than the target department was a particularly important finding. Among the 9525 patients who returned to outpatient care following APTS recall, 90.2% originated from other departments, and this pattern was observed across all included departments. This finding highlights an important challenge in patient management within large general hospitals: clinically relevant abnormalities may be identified in one department, whereas subsequent evaluation or treatment may require care from another specialty. When patient identification and subsequent management are fragmented across departments, gaps may arise as patients move between different parts of the healthcare system. By integrating multisource clinical information and incorporating patient identification, task assignment, recall management, and return-to-care tracking into a unified workflow, APTS may provide a mechanism for identifying patients outside the target department who may require further specialty management and incorporating them into the corresponding management pathway. However, this proportion may partly reflect the predefined patient identification and assignment rules of APTS. Some patients may also have returned to the target department through usual care pathways independently of APTS recall; therefore, these findings do not establish that APTS resolved previously unmet follow-up needs.

Notably, the substantial volume of cross-departmental patient-management activity recorded within the APTS pathway was not accompanied by a significant increase in departmental outpatient volume. The estimated changes in monthly unique outpatients and monthly outpatient visits following APTS implementation were −2.5% (95% CI, −11.2% to 7.0%) and −1.9% (95% CI, −12.1% to 9.5%), respectively, and the event-time analysis likewise provided no evidence of a sustained change. These findings are consistent with the observed cross-departmental pattern of APTS activity. Rather than indicating a simple expansion of outpatient service volume, the findings suggest that APTS may facilitate the identification and management of patients within existing care pathways. However, the absence of a statistically significant change in departmental outpatient volume may also reflect substitution effects, capacity constraints, or care that would have occurred through usual pathways without APTS. Furthermore, the confidence intervals do not exclude potentially meaningful changes in outpatient service utilization. Given the small number of included departments, the precision of effect estimates was limited. Therefore, the observed findings should not be interpreted as evidence that APTS reorganized care rather than expanded outpatient volume.

The operational findings should, however, be interpreted as evidence of the establishment and use of a structured patient management pathway rather than as direct evidence that APTS improved follow-up completion. The primary causal estimand of this study was the effect of APTS implementation on department-level outpatient volume, whereas the APTS operational measures were summarized descriptively to characterize activities recorded within the structured management pathway. The proportion of assigned patients entering recall management ranged from 4.3% to 81.8% across departments. This variation may reflect differences in screening criteria, patient characteristics, and clinical management needs across departments, although the available aggregated data did not allow the contributions of these factors to be determined. Previous studies have evaluated electronic health record-based alerts, automated identification of patients requiring follow-up, and patient outreach interventions to address delays in the management of abnormal test results [21,22,23]. However, because comparable structured records of APTS-related patient management activities were not available before implementation, the post-implementation operational data cannot be interpreted as incremental improvements over the previous management approach. The 9525 patients who returned to outpatient care following APTS recall therefore represent the observed scale of return-to-care activity captured within the APTS pathway, but these data do not establish that APTS increased an individual patient’s probability of returning for follow-up care. This distinction is important when evaluating digital health interventions because greater completeness and traceability of management records do not necessarily indicate corresponding changes in patients’ healthcare-seeking behavior.

From a patient management perspective, the potential role of APTS may be concentrated in the earlier stages of the care pathway. Previous digital health studies have frequently focused on post-discharge follow-up, management of patients lost to follow-up, and prevention of readmissions [24,25], whereas APTS places greater emphasis on connecting the identification of clinically relevant findings with subsequent specialty management. In this context, its potential contribution may lie in reducing gaps between the identification of patients requiring further attention and their incorporation into the appropriate specialty management pathway. Such effects would be expected to emerge first in proximal process measures, including patient identification, task assignment, recall management, and return-to-care tracking, rather than necessarily producing detectable changes in aggregate outpatient volume. However, this study did not directly assess downstream patient-level effectiveness. Future studies should evaluate measurable indicators including successful recall rate, time from recall to return, loss-to-follow-up rate, completion of recommended evaluations, treatment initiation, and clinically relevant patient outcomes to determine whether APTS-supported management translates into improvements in follow-up effectiveness and continuity of care.

This study has several strengths. First, it used longitudinal real-world data from the phased implementation of APTS across multiple clinical departments, thereby reflecting system use within routine hospital workflows. Second, by examining department-level outpatient volume together with APTS operational measures—including patient assignment, recall management, return to outpatient care following recall, and departmental origins of recalled outpatients—the study evaluated implementation from complementary perspectives of service volume and patient management pathways. Third, the Callaway–Sant’Anna staggered difference-in-differences approach was used to account for variation in implementation timing across departments, supplemented by event-time and sensitivity analyses to assess the robustness and temporal pattern of the findings.

Several limitations should be considered. First, this was a single-center study based primarily on aggregated department-month data, which limited patient-level inference and the ability to account for potentially relevant time-varying factors, such as changes in patient case mix, staffing, department-specific service organization, or other concurrent changes. Although the staggered difference-in-differences design used contemporaneous not-yet-treated departments as comparison units and the event-time analysis did not identify statistically significant differential pre-implementation trends, residual confounding from unmeasured department-specific time-varying factors cannot be excluded. Second, comparable structured records of patient identification, recall management, and return-to-care activities were unavailable before APTS implementation; therefore, the 9525 observed return-to-care events describe the operational scale of the APTS pathway rather than additional visits caused by APTS. A consistently defined denominator representing all patients clinically eligible for APTS-supported recall was also unavailable across departments, precluding calculation of standardized rates per 1000 eligible patients. Third, only nine departments were included, and the number of not-yet-treated comparison departments decreased as implementation progressed, limiting statistical precision and precluding definitive confirmation of the parallel trends assumption. Because no not-yet-treated comparison departments remained after September 2025, the difference-in-differences analysis could not identify longer-term post-implementation effects, despite the availability of longitudinal observations through September 2026. Finally, longer-term effects on healthcare utilization, continuity of care, and clinical outcomes could not be adequately evaluated in the present study.

## 5. Conclusions

APTS supported a structured pathway for patient identification, task assignment, recall management, and return-to-care tracking, with 90.2% of patients returning to outpatient care following APTS recall originating from departments other than the target department. Meanwhile, APTS implementation was not associated with significant changes in departmental outpatient volume. These findings suggest that APTS may support the identification and management of patients across departmental boundaries. However, the absence of a statistically significant change in departmental outpatient volume should be interpreted cautiously, as it may reflect substitution effects, capacity constraints, or care that would have occurred independently of APTS.

## Figures and Tables

**Figure 1:**
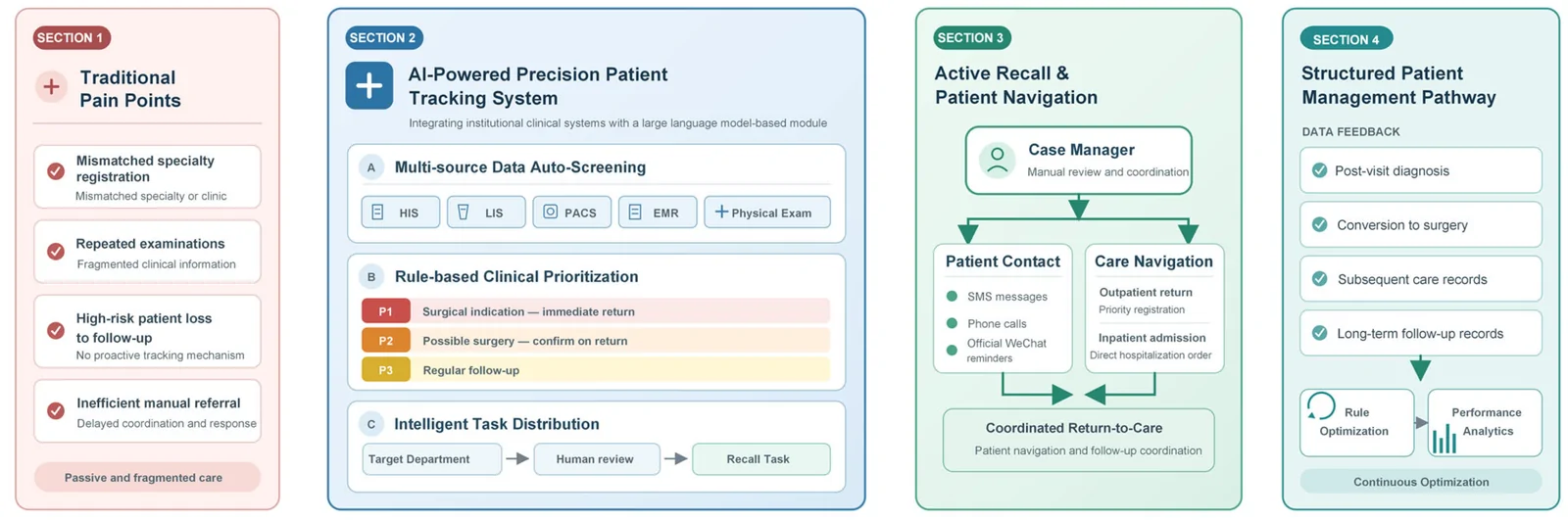
Workflow of APTS. APTS integrates multisource clinical information to support patient identification, task assignment, manual review, recall management, and return-to-care tracking. Patients identified as requiring further clinical attention are assigned to the appropriate target department and case manager for manual review. Patients confirmed as requiring active recall may be contacted through multiple communication channels to facilitate subsequent specialty care. Recall management and return-to-care activities are recorded within the system, forming a structured patient management pathway. Abbreviations: HIS, Hospital Information System; LIS, Laboratory Information System; PACS, Picture Archiving and Communication System; EMR, Electronic Medical Record.

**Figure 2:**
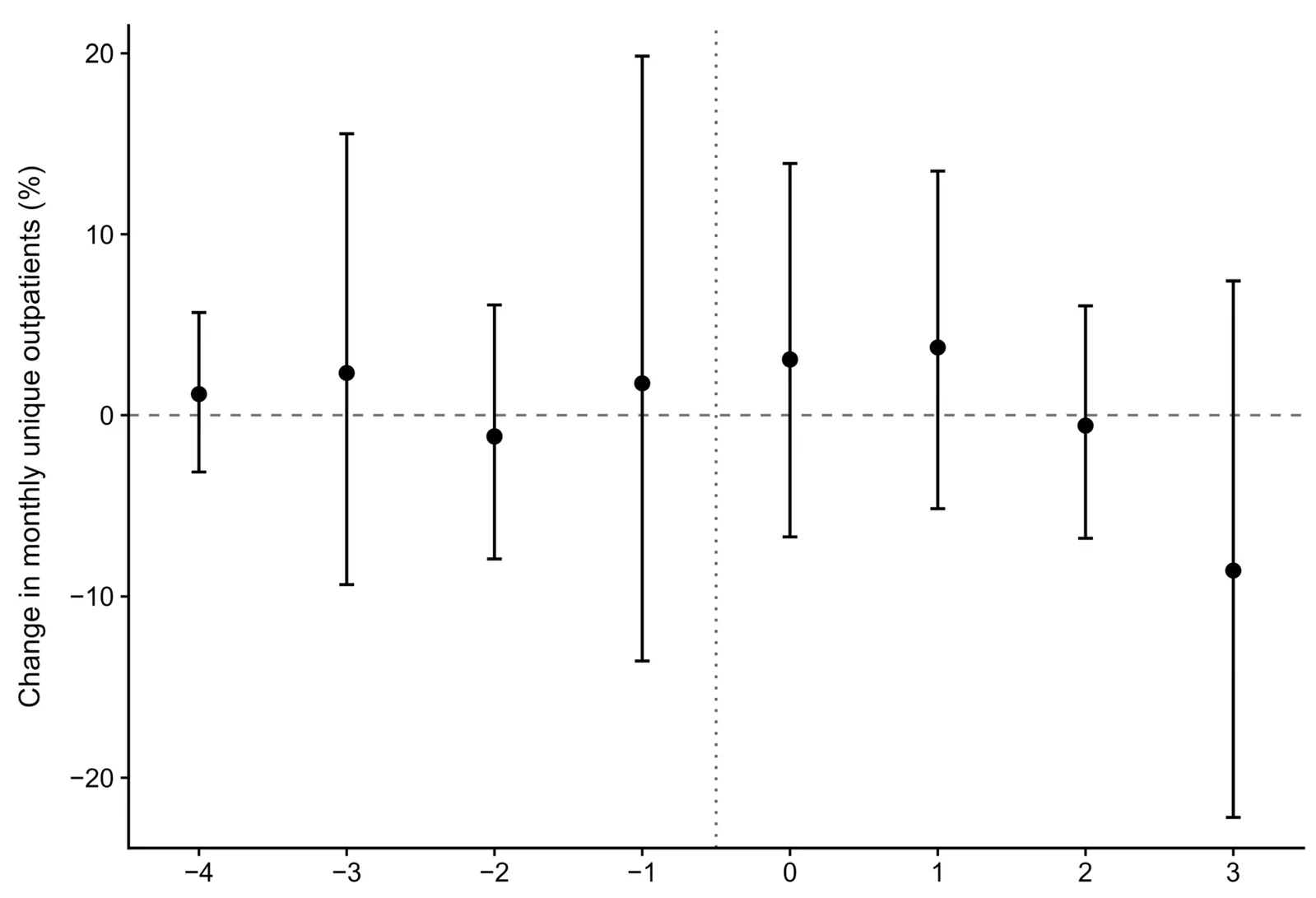
Event-time changes in monthly unique outpatients before and after APTS implementation. Points represent event-time-specific estimates derived from the Callaway–Sant’Anna staggered difference-in-differences analysis, with not-yet-treated departments serving as the comparison group. Outcomes were analyzed on the logarithmic scale and converted to percentage changes using 100 × [exp(ATT) − 1]. Error bars represent 95% simultaneous confidence intervals. Event time 0 represents the month of APTS implementation, and the vertical dashed line separates the pre-implementation and post-implementation periods.

**Figure 3:**
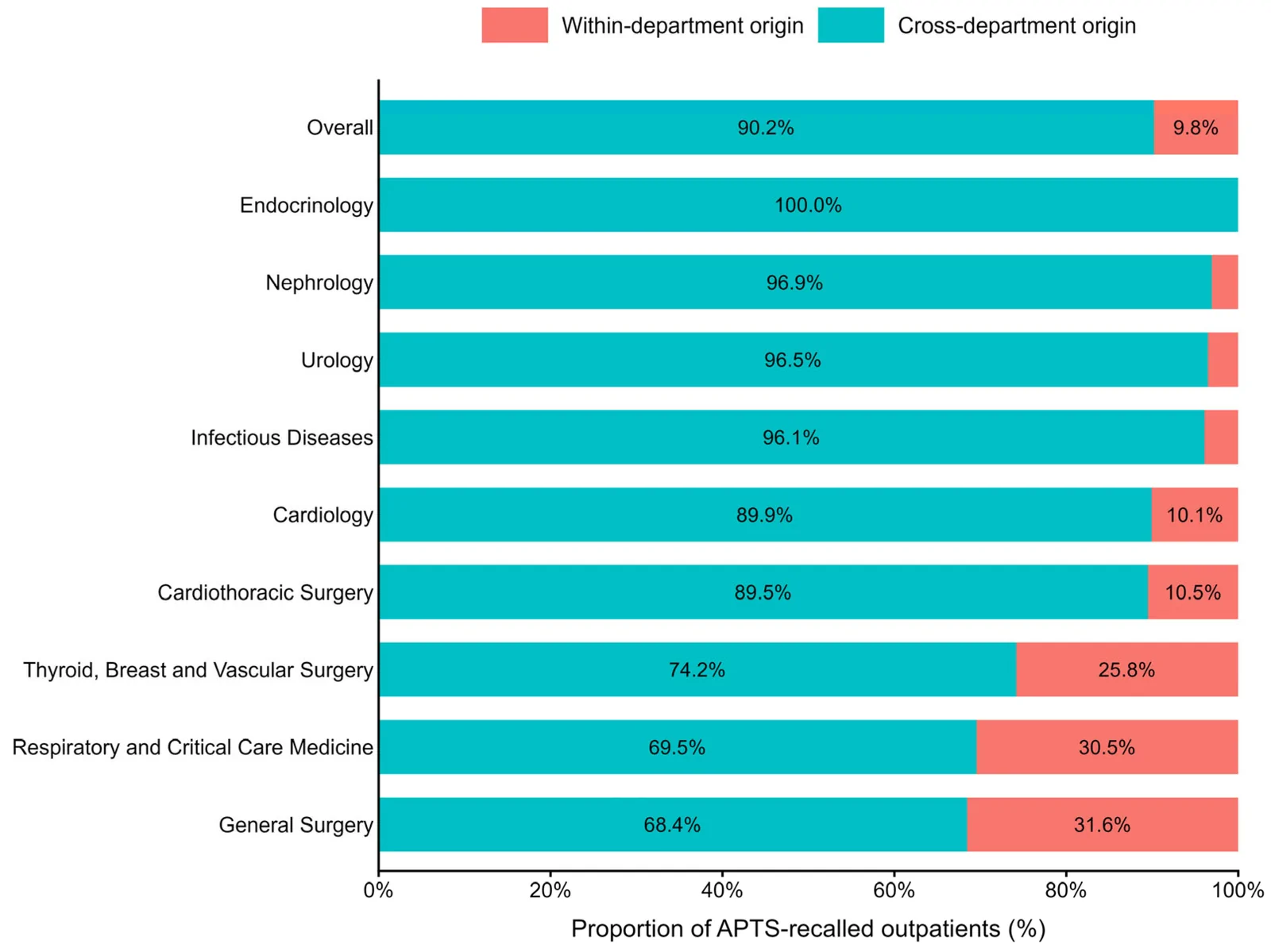
Departmental origins of patients returning to outpatient care following APTS recall. Bars show the distribution of recalled outpatients according to whether they originated from the target department or from other departments. Percentages were calculated from aggregated patient counts for each department over the post-implementation period rather than as averages of monthly percentages. “Overall” represents the aggregated data from all nine departments.

**Table 1:** Department Characteristics and APTS Implementation Timing.

Department	APTS Implementation Month	Pre-Implementation Months	Post-Implementation Months	Monthly Outpatient Visits, Median (IQR)	Monthly Unique Outpatients, Median (IQR)
Cardiothoracic Surgery	March 2025	2	19	161 (155–177)	156 (150–167)
General Surgery	March 2025	2	19	4532 (4069–5208)	4226 (3791–4892)
Respiratory and Critical Care Medicine	April 2025	3	18	3439 (3263–3884)	3113 (2919–3485)
Thyroid, Breast, and Vascular Surgery	April 2025	3	18	1421 (1374–1496)	1299 (1269–1364)
Urology	July 2025	6	15	2500 (2414–2666)	2186 (2113–2331)
Cardiology	August 2025	7	14	3698 (3438–4388)	3509 (3244–4099)
Endocrinology	September 2025	8	13	343 (309–376)	304 (279–334)
Infectious Diseases	October 2025	9	12	2848 (2691–3063)	2590 (2429–2801)
Nephrology	October 2025	9	12	1964 (1841–2367)	1800 (1694–2141)

Note: Continuous variables are presented as medians (interquartile ranges [IQRs]) based on department-month observations over the study period (January 2025 to September 2026). The month of APTS implementation was classified as the first post-implementation month.

**Table 2:** Changes in Department-Level Outpatient Volume Following APTS Implementation.

Outcome	ATT (Log Scale)	Relative Change, %	95% CI
Monthly Unique Outpatients	−0.0256	−2.5	−11.2 to 7.0
Monthly Outpatient Visits	−0.0190	−1.9	−12.1 to 9.5

Note: Estimates were obtained using the Callaway–Sant’Anna staggered difference-in-differences approach, with not-yet-treated departments serving as the comparison group. Both outcomes were log-transformed. Relative changes were calculated as 100 × [exp(ATT) − 1]. ATT, average treatment effect on the treated; CI, confidence interval.

**Table 3:** Patient Management Following APTS Implementation by Department.

Department	Post-Implementation Months	Patients Assigned by APTS	Patients Entering Recall Management	Proportion Entering Recall Management (%)	Patients Returning to Outpatient Care Following APTS Recall	Recall–Return Rate (%)
Cardiothoracic Surgery	19	5733	1453	25.3	219	15.1
General Surgery	19	6231	646	10.4	187	28.9
Respiratory and Critical Care Medicine	18	19,000	818	4.3	197	24.1
Thyroid, Breast, and Vascular Surgery	18	7771	6357	81.8	2296	36.1
Urology	15	15,829	10,107	63.9	1697	16.8
Cardiology	14	5796	559	9.6	109	19.5
Endocrinology	13	8041	6087	75.7	1001	16.4
Infectious Diseases	12	6579	4820	73.3	967	20.1
Nephrology	12	34,640	19,582	56.5	2852	14.6
Total	140	109,620	50,429	46.0	9525	18.9

Note: Data include observations from the month of APTS implementation onward for each department. The proportion entering recall management was calculated as the number of patients entering recall management divided by the number of patients assigned by APTS. The recall–return rate was calculated as the number of patients returning to outpatient care following APTS recall divided by the number of patients entering recall management. Overall percentages were calculated from aggregated patient counts rather than as averages of department- or month-specific percentages.

## Data Availability

The department-level monthly dataset used in this study is provided in Supplementary Table S3. The dataset includes aggregated outpatient service and APTS-related patient management data from nine clinical departments between January 2025 and September 2026. No patient-level identifiable information is included.

## Supplementary Materials

The following supporting information can be downloaded at: https://www.mdpi.com/article/10.3390/healthcare14213586/s1, Table S1: Source composition of APTS-recalled outpatients across departments; Table S2: Sensitivity analysis of changes in department-level outpatient volume using outcomes on the original count scale; Table S3: Department-level monthly data used for the longitudinal evaluation of the AI-powered Precision Patient Tracking System (APTS).
