# Supplementary material for: An AI-Powered Precision Patient Tracking System for Interdepartmental Patient Identification and Recall: A Real-World Longitudinal Study
Source: Healthcare (Basel). 2026 Oct 23;14(21):3586. doi: 10.3390/healthcare14213586 (PMC13762980; doi:10.3390/healthcare14213586)
Supplement: Supplementary file 1 [file healthcare-14-03586-s001.zip › healthcare-4573272-supplementary.pdf]

Supplementary Table S1. Source composition of APTS-recalled outpatients across departments

**Department**	**Total APTS-recalled outpatients**	**Within-department origin, n (%)**	**Cross-department origin, n (%)**
Cardiothoracic Surgery	219	23 (10.5)	196 (89.5)
General Surgery	187	59 (31.6)	128 (68.4)
Respiratory and Critical Care Medicine	197	60 (30.5)	137 (69.5)
Thyroid, Breast and Vascular Surgery	2,296	593 (25.8)	1,703 (74.2)
Urology	1,697	60 (3.5)	1,637 (96.5)
Cardiology	109	11 (10.1)	98 (89.9)
Endocrinology	1,001	0 (0.0)	1,001 (100.0)
Infectious Diseases	967	38 (3.9)	929 (96.1)
Nephrology	2,852	88 (3.1)	2,764 (96.9)
**Overall**	**9,525**	**932 (9.8)**	**8,593 (90.2)**

Notes: Data include APTS-recalled outpatients from the month of APTS implementation onward. Within-department origin refers to APTS-recalled outpatients originating from the target department. Cross-department origin refers to APTS-recalled outpatients originating from departments other than the target department. Percentages were calculated from pooled patient counts over the post-implementation period for each department rather than by averaging monthly percentages. Overall percentages were calculated from pooled counts across all nine departments.

Supplementary Table S2. Sensitivity analysis of changes in department-level outpatient volume using outcomes on the original count scale

Outcome	ATT, count scale	SE	95% CI
Monthly unique outpatients	**56.63**	234.87	**−403.71 to 516.97**
Monthly outpatient encounters	**73.13**	267.24	**−450.65 to 596.91**

Notes: Estimates were obtained using the Callaway-Sant'Anna staggered difference-in-differences estimator, with not-yet-treated departments serving as controls. Outcomes were analyzed on their original count scales without logarithmic transformation. ATT indicates average treatment effect on the treated; SE, standard error; CI, confidence interval.

Supplementary Table S3. Department-level monthly data used for the longitudinal evaluation of the AI-powered Precision Patient Tracking System (APTS)

Year-Month	Clinical Department	APTS Implementation Month	Event Time	Patients Assigned by APTS	Patients Entering Recall Management	Patients Returning to Outpatient Care Following APTS Recall	Recalled Outpatients Originating from the Target Department	Recalled Outpatients Originating from Other Departments	Monthly Outpatient Visits	Monthly Unique Outpatients
2025-01	Infectious Diseases	2025-10	-9	0	0	0	0	0	2585	2359
2025-02	Infectious Diseases	2025-10	-8	0	0	0	0	0	2144	1974
2025-03	Infectious Diseases	2025-10	-7	0	0	0	0	0	2781	2499
2025-04	Infectious Diseases	2025-10	-6	0	0	0	0	0	2551	2316
2025-05	Infectious Diseases	2025-10	-5	0	0	0	0	0	2760	2506
2025-06	Infectious Diseases	2025-10	-4	0	0	0	0	0	2574	2335
2025-07	Infectious Diseases	2025-10	-3	0	0	0	0	0	2691	2429
2025-08	Infectious Diseases	2025-10	-2	0	0	0	0	0	2819	2555
2025-09	Infectious Diseases	2025-10	-1	0	0	0	0	0	2726	2486
2025-10	Infectious Diseases	2025-10	0	166	148	48	33	15	2848	2590
2025-11	Infectious Diseases	2025-10	1	764	598	124	5	119	2925	2672
2025-12	Infectious Diseases	2025-10	2	634	600	131	0	131	3182	2895
2026-01	Infectious Diseases	2025-10	3	529	500	94	0	94	3158	2868
2026-02	Infectious Diseases	2025-10	4	314	216	38	0	38	1994	1870

Year-Month	Clinical Department	APTS Implementation Month	Event Time	Patients Assigned by APTS	Patients Entering Recall Management	Patients Returning to Outpatient Care Following APTS Recall	Recalled Outpatients Originating from the Target Department	Recalled Outpatients Originating from Other Departments	Monthly Outpatient Visits	Monthly Unique Outpatients
2026-03	Infectious Diseases	2025-10	5	843	683	139	0	139	2896	2650
2026-04	Infectious Diseases	2025-10	6	755	472	92	0	92	2907	2632
2026-05	Infectious Diseases	2025-10	7	840	502	107	0	107	3063	2801
2026-06	Infectious Diseases	2025-10	8	794	435	77	0	77	3106	2869
2026-07	Infectious Diseases	2025-10	9	388	221	32	0	32	3141	2903
2026-08	Infectious Diseases	2025-10	10	296	239	53	0	53	3217	2973
2026-09	Infectious Diseases	2025-10	11	256	206	32	0	32	2986	2784
2025-01	Respiratory and Critical Care Medicine	2025-04	-3	0	0	0	0	0	3509	3173
2025-02	Respiratory and Critical Care Medicine	2025-04	-2	0	0	0	0	0	3419	3104
2025-03	Respiratory and Critical Care Medicine	2025-04	-1	0	0	0	0	0	3439	3113
2025-04	Respiratory and Critical Care Medicine	2025-04	0	1526	58	10	0	10	3869	3482
2025-05	Respiratory and Critical Care Medicine	2025-04	1	1351	71	17	7	10	4150	3690
2025-06	Respiratory	2025-04	2	1201	88	22	18	4	3263	2919

Year-Month	Clinical Department	APTS Implementation Month	Event Time	Patients Assigned by APTS	Patients Entering Recall Management	Patients Returning to Outpatient Care Following APTS Recall	Recalled Outpatients Originating from the Target Department	Recalled Outpatients Originating from Other Departments	Monthly Outpatient Visits	Monthly Unique Outpatients
	and Critical Care Medicine									
2025-07	Respiratory and Critical Care Medicine	2025-04	3	1062	81	27	18	9	2797	2523
2025-08	Respiratory and Critical Care Medicine	2025-04	4	1421	102	29	17	12	2741	2479
2025-09	Respiratory and Critical Care Medicine	2025-04	5	1484	110	20	0	20	2737	2458
2025-10	Respiratory and Critical Care Medicine	2025-04	6	2002	54	12	0	12	2889	2608
2025-11	Respiratory and Critical Care Medicine	2025-04	7	1242	79	18	0	18	3623	3280
2025-12	Respiratory and Critical Care Medicine	2025-04	8	1267	60	11	0	11	4453	3984
2026-01	Respiratory and Critical Care Medicine	2025-04	9	624	19	4	0	4	4098	3652
2026-02	Respiratory and Critical Care Medicine	2025-04	10	448	17	2	0	2	2690	2469
2026-03	Respiratory and Critical Care Medicine	2025-04	11	693	16	8	0	8	3272	2958
2026-04	Respiratory	2025-04	12	692	28	7	0	7	3392	3068

Year-Month	Clinical Department	APTS Implementation Month	Event Time	Patients Assigned by APTS	Patients Entering Recall Management	Patients Returning to Outpatient Care Following APTS Recall	Recalled Outpatients Originating from the Target Department	Recalled Outpatients Originating from Other Departments	Monthly Outpatient Visits	Monthly Unique Outpatients
	and Critical Care Medicine									
2026-05	Respiratory and Critical Care Medicine	2025-04	13	707	7	1	0	1	3884	3485
2026-06	Respiratory and Critical Care Medicine	2025-04	14	828	17	3	0	3	3935	3551
2026-07	Respiratory and Critical Care Medicine	2025-04	15	1120	7	4	0	4	4324	3867
2026-08	Respiratory and Critical Care Medicine	2025-04	16	1052	4	2	0	2	3529	3189
2026-09	Respiratory and Critical Care Medicine	2025-04	17	280	0	0	0	0	3341	3009
2025-01	Thyroid, Breast and Vascular Surgery	2025-04	-3	0	0	0	0	0	1261	1144
2025-02	Thyroid, Breast and Vascular Surgery	2025-04	-2	0	0	0	0	0	1077	1022
2025-03	Thyroid, Breast and Vascular Surgery	2025-04	-1	0	0	0	0	0	1587	1479
2025-04	Thyroid, Breast and Vascular Surgery	2025-04	0	157	149	66	17	49	1518	1428
2025-05	Thyroid, Breast	2025-04	1	147	146	53	34	19	1536	1397

Year-Month	Clinical Department	APTS Implementation Month	Event Time	Patients Assigned by APTS	Patients Entering Recall Management	Patients Returning to Outpatient Care Following APTS Recall	Recalled Outpatients Originating from the Target Department	Recalled Outpatients Originating from Other Departments	Monthly Outpatient Visits	Monthly Unique Outpatients
	and Vascular Surgery									
2025-06	Thyroid, Breast and Vascular Surgery	2025-04	2	162	158	54	46	8	1526	1410
2025-07	Thyroid, Breast and Vascular Surgery	2025-04	3	458	439	170	16	154	1444	1339
2025-08	Thyroid, Breast and Vascular Surgery	2025-04	4	511	474	171	62	109	1412	1312
2025-09	Thyroid, Breast and Vascular Surgery	2025-04	5	220	202	75	14	61	1319	1200
2025-10	Thyroid, Breast and Vascular Surgery	2025-04	6	514	355	150	54	96	1400	1299
2025-11	Thyroid, Breast and Vascular Surgery	2025-04	7	629	617	225	62	163	1325	1224
2025-12	Thyroid, Breast and Vascular Surgery	2025-04	8	474	429	168	58	110	1492	1348
2026-01	Thyroid, Breast and Vascular Surgery	2025-04	9	329	323	128	44	84	1432	1289
2026-02	Thyroid, Breast and Vascular Surgery	2025-04	10	237	228	61	11	50	857	798
2026-03	Thyroid, Breast	2025-04	11	379	371	137	39	98	1484	1364

Year-Month	Clinical Department	APTS Implementation Month	Event Time	Patients Assigned by APTS	Patients Entering Recall Management	Patients Returning to Outpatient Care Following APTS Recall	Recalled Outpatients Originating from the Target Department	Recalled Outpatients Originating from Other Departments	Monthly Outpatient Visits	Monthly Unique Outpatients
	and Vascular Surgery									
2026-04	Thyroid, Breast and Vascular Surgery	2025-04	12	453	395	125	35	90	1374	1269
2026-05	Thyroid, Breast and Vascular Surgery	2025-04	13	608	430	165	17	148	1418	1287
2026-06	Thyroid, Breast and Vascular Surgery	2025-04	14	564	408	144	20	124	1398	1282
2026-07	Thyroid, Breast and Vascular Surgery	2025-04	15	605	438	156	23	133	1421	1290
2026-08	Thyroid, Breast and Vascular Surgery	2025-04	16	661	359	128	23	105	1549	1400
2026-09	Thyroid, Breast and Vascular Surgery	2025-04	17	663	436	120	18	102	1496	1353
2025-01	Urology	2025-07	-6	0	0	0	0	0	1840	1588
2025-02	Urology	2025-07	-5	0	0	0	0	0	1756	1597
2025-03	Urology	2025-07	-4	0	0	0	0	0	2433	2150
2025-04	Urology	2025-07	-3	0	0	0	0	0	2341	2092
2025-05	Urology	2025-07	-2	0	0	0	0	0	2498	2126
2025-06	Urology	2025-07	-1	0	0	0	0	0	2442	2139
2025-07	Urology	2025-07	0	200	136	26	20	6	2619	2268
2025-08	Urology	2025-07	1	474	406	70	39	31	2579	2223
2025-09	Urology	2025-07	2	1125	838	154	1	153	2414	2113
2025-10	Urology	2025-07	3	1425	934	160	0	160	2589	2262

Year-Month	Clinical Department	APTS Implementation Month	Event Time	Patients Assigned by APTS	Patients Entering Recall Management	Patients Returning to Outpatient Care Following APTS Recall	Recalled Outpatients Originating from the Target Department	Recalled Outpatients Originating from Other Departments	Monthly Outpatient Visits	Monthly Unique Outpatients
2025-11	Urology	2025-07	4	1415	905	149	0	149	2576	2218
2025-12	Urology	2025-07	5	1115	481	80	0	80	2500	2186
2026-01	Urology	2025-07	6	1045	261	53	0	53	2477	2119
2026-02	Urology	2025-07	7	595	404	51	0	51	1586	1427
2026-03	Urology	2025-07	8	1147	958	105	0	105	2348	2054
2026-04	Urology	2025-07	9	1130	836	142	0	142	2666	2331
2026-05	Urology	2025-07	10	1159	799	131	0	131	2786	2415
2026-06	Urology	2025-07	11	1135	764	138	0	138	2671	2338
2026-07	Urology	2025-07	12	1153	757	144	0	144	3475	3008
2026-08	Urology	2025-07	13	1332	813	151	0	151	3117	2699
2026-09	Urology	2025-07	14	1379	815	143	0	143	2876	2560
2025-01	Endocrinology	2025-09	-8	0	0	0	0	0	283	260
2025-02	Endocrinology	2025-09	-7	0	0	0	0	0	237	218
2025-03	Endocrinology	2025-09	-6	0	0	0	0	0	343	304
2025-04	Endocrinology	2025-09	-5	0	0	0	0	0	292	270
2025-05	Endocrinology	2025-09	-4	0	0	0	0	0	311	279
2025-06	Endocrinology	2025-09	-3	0	0	0	0	0	333	303
2025-07	Endocrinology	2025-09	-2	0	0	0	0	0	309	282
2025-08	Endocrinology	2025-09	-1	0	0	0	0	0	314	290
2025-09	Endocrinology	2025-09	0	15	8	2	0	2	307	271
2025-10	Endocrinology	2025-09	1	129	76	9	0	9	315	291
2025-11	Endocrinology	2025-09	2	931	736	142	0	142	354	322
2025-12	Endocrinology	2025-09	3	684	513	83	0	83	353	315
2026-01	Endocrinology	2025-09	4	830	651	113	0	113	376	334
2026-02	Endocrinology	2025-09	5	400	314	34	0	34	271	243
2026-03	Endocrinology	2025-09	6	867	648	109	0	109	390	352
2026-04	Endocrinology	2025-09	7	751	548	92	0	92	386	339
2026-05	Endocrinology	2025-09	8	767	524	82	0	82	398	360
2026-06	Endocrinology	2025-09	9	696	573	89	0	89	357	327

Year-Month	Clinical Department	APTS Implementation Month	Event Time	Patients Assigned by APTS	Patients Entering Recall Management	Patients Returning to Outpatient Care Following APTS Recall	Recalled Outpatients Originating from the Target Department	Recalled Outpatients Originating from Other Departments	Monthly Outpatient Visits	Monthly Unique Outpatients
2026-07	Endocrinology	2025-09	10	607	428	79	0	79	352	310
2026-08	Endocrinology	2025-09	11	696	542	92	0	92	377	336
2026-09	Endocrinology	2025-09	12	668	526	75	0	75	384	343
2025-01	General Surgery	2025-03	-2	0	0	0	0	0	3354	3171
2025-02	General Surgery	2025-03	-1	0	0	0	0	0	2745	2626
2025-03	General Surgery	2025-03	0	565	12	7	1	6	3935	3650
2025-04	General Surgery	2025-03	1	1188	14	5	0	5	3952	3692
2025-05	General Surgery	2025-03	2	613	26	8	7	1	4069	3796
2025-06	General Surgery	2025-03	3	487	28	7	7	0	4070	3791
2025-07	General Surgery	2025-03	4	627	32	12	11	1	4211	3903
2025-08	General Surgery	2025-03	5	625	68	23	19	4	4464	4123
2025-09	General Surgery	2025-03	6	190	42	10	0	10	4132	3829
2025-10	General Surgery	2025-03	7	161	42	10	0	10	4532	4226
2025-11	General Surgery	2025-03	8	198	67	10	0	10	4609	4292
2025-12	General Surgery	2025-03	9	186	57	17	1	16	4697	4344
2026-01	General Surgery	2025-03	10	153	41	11	2	9	5099	4724
2026-02	General	2025-03	11	110	15	4	2	2	3368	3210

Year-Month	Clinical Department	APTS Implementation Month	Event Time	Patients Assigned by APTS	Patients Entering Recall Management	Patients Returning to Outpatient Care Following APTS Recall	Recalled Outpatients Originating from the Target Department	Recalled Outpatients Originating from Other Departments	Monthly Outpatient Visits	Monthly Unique Outpatients
	Surgery									
2026-03	General Surgery	2025-03	12	181	51	18	2	16	5273	4892
2026-04	General Surgery	2025-03	13	155	30	11	4	7	5189	4811
2026-05	General Surgery	2025-03	14	154	28	10	1	9	5667	5260
2026-06	General Surgery	2025-03	15	167	35	8	1	7	5208	4907
2026-07	General Surgery	2025-03	16	150	16	5	0	5	5615	5226
2026-08	General Surgery	2025-03	17	153	15	3	0	3	5794	5385
2026-09	General Surgery	2025-03	18	168	27	8	1	7	5694	5340
2025-01	Nephrology	2025-10	-9	0	0	0	0	0	1616	1467
2025-02	Nephrology	2025-10	-8	0	0	0	0	0	1355	1274
2025-03	Nephrology	2025-10	-7	0	0	0	0	0	1964	1800
2025-04	Nephrology	2025-10	-6	0	0	0	0	0	1774	1634
2025-05	Nephrology	2025-10	-5	0	0	0	0	0	1904	1735
2025-06	Nephrology	2025-10	-4	0	0	0	0	0	1886	1731
2025-07	Nephrology	2025-10	-3	0	0	0	0	0	1834	1694
2025-08	Nephrology	2025-10	-2	0	0	0	0	0	1841	1682
2025-09	Nephrology	2025-10	-1	0	0	0	0	0	1872	1718
2025-10	Nephrology	2025-10	0	1232	533	69	2	67	1910	1735
2025-11	Nephrology	2025-10	1	3070	1895	272	17	255	2041	1856
2025-12	Nephrology	2025-10	2	2471	1387	201	19	182	2105	1898
2026-01	Nephrology	2025-10	3	3526	2736	384	22	362	2367	2120
2026-02	Nephrology	2025-10	4	1761	905	94	3	91	1620	1483

Year-Month	Clinical Department	APTS Implementation Month	Event Time	Patients Assigned by APTS	Patients Entering Recall Management	Patients Returning to Outpatient Care Following APTS Recall	Recalled Outpatients Originating from the Target Department	Recalled Outpatients Originating from Other Departments	Monthly Outpatient Visits	Monthly Unique Outpatients
2026-03	Nephrology	2025-10	5	3973	2502	347	21	326	2301	2092
2026-04	Nephrology	2025-10	6	3204	1691	275	1	274	2390	2179
2026-05	Nephrology	2025-10	7	3297	1765	272	3	269	2485	2267
2026-06	Nephrology	2025-10	8	3141	1539	248	0	248	2483	2252
2026-07	Nephrology	2025-10	9	2933	1405	234	0	234	2385	2190
2026-08	Nephrology	2025-10	10	3158	1658	258	0	258	2524	2287
2026-09	Nephrology	2025-10	11	2874	1566	198	0	198	2361	2141
2025-01	Cardiothoracic Surgery	2025-03	-2	0	0	0	0	0	117	113
2025-02	Cardiothoracic Surgery	2025-03	-1	0	0	0	0	0	159	152
2025-03	Cardiothoracic Surgery	2025-03	0	52	48	11	0	11	194	188
2025-04	Cardiothoracic Surgery	2025-03	1	436	191	29	6	23	177	167
2025-05	Cardiothoracic Surgery	2025-03	2	226	63	12	4	8	190	183
2025-06	Cardiothoracic Surgery	2025-03	3	150	49	1	0	1	161	151
2025-07	Cardiothoracic Surgery	2025-03	4	141	43	5	2	3	162	157
2025-08	Cardiothoracic Surgery	2025-03	5	253	68	7	5	2	181	175
2025-09	Cardiothoracic Surgery	2025-03	6	378	147	22	1	21	159	151
2025-10	Cardiothoracic Surgery	2025-03	7	291	124	13	0	13	169	156
2025-11	Cardiothoracic Surgery	2025-03	8	295	77	12	0	12	185	180
2025-12	Cardiothoracic	2025-03	9	400	59	15	1	14	185	179

Year-Month	Clinical Department	APTS Implementation Month	Event Time	Patients Assigned by APTS	Patients Entering Recall Management	Patients Returning to Outpatient Care Following APTS Recall	Recalled Outpatients Originating from the Target Department	Recalled Outpatients Originating from Other Departments	Monthly Outpatient Visits	Monthly Unique Outpatients
	Surgery									
2026-01	Cardiothoracic Surgery	2025-03	10	412	43	8	2	6	168	160
2026-02	Cardiothoracic Surgery	2025-03	11	286	44	5	0	5	104	97
2026-03	Cardiothoracic Surgery	2025-03	12	441	66	9	0	9	173	161
2026-04	Cardiothoracic Surgery	2025-03	13	386	79	9	0	9	155	151
2026-05	Cardiothoracic Surgery	2025-03	14	358	106	18	1	17	144	138
2026-06	Cardiothoracic Surgery	2025-03	15	278	69	10	0	10	142	138
2026-07	Cardiothoracic Surgery	2025-03	16	311	62	14	0	14	154	150
2026-08	Cardiothoracic Surgery	2025-03	17	327	61	8	0	8	158	147
2026-09	Cardiothoracic Surgery	2025-03	18	312	54	11	1	10	161	157
2025-01	Cardiology	2025-08	-7	0	0	0	0	0	3104	2948
2025-02	Cardiology	2025-08	-6	0	0	0	0	0	2663	2536
2025-03	Cardiology	2025-08	-5	0	0	0	0	0	3698	3509
2025-04	Cardiology	2025-08	-4	0	0	0	0	0	3438	3246
2025-05	Cardiology	2025-08	-3	0	0	0	0	0	3316	3115
2025-06	Cardiology	2025-08	-2	0	0	0	0	0	3364	3199
2025-07	Cardiology	2025-08	-1	0	0	0	0	0	3468	3244
2025-08	Cardiology	2025-08	0	59	44	14	9	5	3444	3245
2025-09	Cardiology	2025-08	1	59	41	16	0	16	3502	3290
2025-10	Cardiology	2025-08	2	49	13	4	0	4	3497	3275

Year-Month	Clinical Department	APTS Implementation Month	Event Time	Patients Assigned by APTS	Patients Entering Recall Management	Patients Returning to Outpatient Care Following APTS Recall	Recalled Outpatients Originating from the Target Department	Recalled Outpatients Originating from Other Departments	Monthly Outpatient Visits	Monthly Unique Outpatients
2025-11	Cardiology	2025-08	3	113	21	5	1	4	3779	3565
2025-12	Cardiology	2025-08	4	357	111	20	1	19	4031	3774
2026-01	Cardiology	2025-08	5	494	92	15	0	15	4534	4215
2026-02	Cardiology	2025-08	6	394	62	6	0	6	2883	2740
2026-03	Cardiology	2025-08	7	545	125	18	0	18	4539	4230
2026-04	Cardiology	2025-08	8	636	27	6	0	6	4763	4435
2026-05	Cardiology	2025-08	9	687	7	4	0	4	4412	4105
2026-06	Cardiology	2025-08	10	706	4	1	0	1	4089	3852
2026-07	Cardiology	2025-08	11	681	1	0	0	0	4356	4069
2026-08	Cardiology	2025-08	12	792	10	0	0	0	4388	4099
2026-09	Cardiology	2025-08	13	224	1	0	0	0	4548	4244

Note: The table presents department-level monthly data from nine clinical departments between January 2025 and September 2026, comprising 189 department-month observations. Year-Month indicates the calendar month of observation. APTS Implementation Month indicates the first month in which APTS was formally implemented in each department. Event Time is defined relative to the implementation month, with 0 representing the month of implementation, negative values representing pre-implementation months, and positive values representing post-implementation months. Patients Assigned by APTS refers to patients assigned by APTS for subsequent departmental management. Patients Entering Recall Management refers to assigned patients who subsequently entered the recall management process. Patients Returning to Outpatient Care Following APTS Recall refers to patients who returned to the assigned target department after recall management and before the data extraction cutoff, as identified by linking APTS recall management records with outpatient records using a unique patient identifier that remained consistent across outpatient encounters. No fixed follow-up window was imposed. The source department refers to the department where the patient was initially identified by APTS, whereas the target department refers to the department assigned for subsequent management and recall. Recalled Outpatients Originating from the Target Department refers to patients recalled and subsequently returning to the same department in which they were initially identified. Recalled Outpatients Originating from Other Departments refers to patients initially identified in another department, recalled by the assigned target department, and subsequently returning to that target department. Monthly Outpatient Visits represents the total number of outpatient visits per department per month, whereas Monthly Unique Outpatients represents the number of unique patients receiving outpatient care in each department per month. For pre-implementation months, APTS-specific variables were coded as 0 because APTS-related structured management activities were not yet available; these values should not be interpreted as indicating the absence of patients requiring further management.
